# Methylenetetrahydrofolate reductase C677T polymorphism in patients with gastric and colorectal cancer in a Korean population

**DOI:** 10.1186/1471-2407-10-236

**Published:** 2010-05-26

**Authors:** Lian-Hua Cui, Min-Ho Shin, Sun-Seog Kweon, Hee Nam Kim, Hye-Rim Song, Jin-Mei Piao, Jin-Su Choi, Hyun Jeong Shim, Jun Eul Hwang, Hyeong-Rok Kim, Young-Kyu Park, Soo-Hyun Kim

**Affiliations:** 1 Department of Public Health, Qingdao University Medical college, Qingdao, China; 2 Department of Preventive Medicine, Chonnam National University Medical School, Gwangju, South Korea; 3 Jeonnam Regional Cancer Center, Chonnam National University Hwasun Hospital, Hwasun, Jeollanam-do, South Korea; 4 Genome Research Center for Hematopoietic Diseases, Chonnam National University Hwasun Hospital, Hwasun, Jeollanam-do, South Korea; 5 Department of Hematology-Oncology, Chonnam National University Hwasun Hospital, Hwasun, Jeollanam-do, South Korea; 6 Department of Surgery, Chonnam National University Hwasun Hospital, Hwasun, Jeollanam-do, South Korea; 7 Department of Laboratory Medicine, Chonnam National University Medical School, Gwangju, South Korea

## Abstract

**Background:**

This study was designed to investigate an association between the methylenetetrahydrofolate reductase (MTHFR) C677T polymorphism and the risk of gastric and colorectal cancer in the Korean population.

**Methods:**

We conducted a population-based large-scale case-control study involving 2,213 patients with newly diagnosed gastric cancer, 1,829 patients with newly diagnosed colorectal cancer, and 1,700 healthy controls. Genotyping was performed with peripheral blood DNA for MTHFR C677T polymorphisms. The statistical significance was estimated by logistic regression analysis.

**Results:**

The MTHFR C677T frequencies of CC, CT, and TT genotypes were 35.2%, 47.5%, and 17.3% among stomach cancer, 34%, 50.5%, and 15.5% in colorectal cancer, and 31.8%, 50.7%, and 17.5% in the controls, respectively. The MTHFR 677TT genotype showed a weak opposite association with colorectal cancer compared to the homozygous CC genotype [adjusted age and sex odds ratio (OR) = 0.792, 95% confidence interval (CI) = 0.638-0.984, *P *= 0.035]. Subjects with the MTHFR 677CT showed a significantly reduced risk of gastric cancer compared whose with the 677CC genotype (age- and sex-adjusted OR = 0.810; 95% CI = 0.696-0.942, *P *= 0.006). We also observed no significant interactions between the MTHFR C677T polymorphism and smoking or drinking in the risk of gastric and colorectal cancer.

**Conclusions:**

The T allele was found to provide a weak protective association with gastric cancer and colorectal cancer.

## Background

Although gastric cancer incidence and mortality have been decreasing around the world, it is still the most common cause of cancer death in Korea for both sexes [1]. Colorectal cancer is very common and has increased rapidly along with the westernization of lifestyle in Korea. Although Helicobacter pylori (***H. pylori***) strains have been proposed to be a major cause of gastric cancer, they do not provide a complete explanation. Epidemio- logical studies have indicated an association between folate intake and a reduced risk of certain cancers [2-5], including gastric cancer [4] and colorectal cancer [3]. Folate deficiencies may result in abnormal DNA methylation and uncontrolled gene expression leading to malignant transformation [6,7].

Methylenetetrahydrofolate reductase (MTHFR) is an enzyme that plays an essential role in the metabolism of folic acid and catalyzes the irreversible reduction of 5,10-methylenetetrahydrofolate to 5-methyltetrahydrofolate. A change of C to T at nucleotide 677 in MTHFR C677T results in an amino acid substance change of an alanine to valine, and this substance is associated with reduced enzyme activity that leads to reduced plasma folate levels [8]. Low enzyme activity of MTHFR C677T variant genotypes are associated with DNA hypomethylation, which may induce genomic instability and thereby affect the expression of oncogenes or tumor suppressor genes.

The association between the MTHFRC677T gene polymorphisms and genetic susceptibility to stomach cancer and colorectal cancer has been widely evaluated in recent studies, but with controversial conclusions. Several studies reported that a homozygous variant genotype of the polymorphism of MTHFRC677T was associated with an increased risk of gastric cancer [9,10] and colorectal cancer [11,12]. However, other studies reported that individuals with the MTHFR 677TT genotype had a decreased risk of colorectal cancer[13], whereas yet others observed no association between the MTHFRC677T genotype and genetic susceptibility to gastric and colorectal cancer [14-17]. Considering the studies provided to date, quite inconsistent results have been reported on the association of MTHFR C677T gene polymorphisms with genetic susceptibility to stomach cancer and colorectal cancer. We designed a large-scale population-based case-control study in Korea to evaluate the potential role of the MTHFR C677T gene polymorphism in gastric and colorectal cancer risk, which would help us to screen, treat, survey, and prevent gastric and colorectal cancer.

## Methods

### Subjects

The study population consists of 2,213 patients with newly diagnosed gastric cancer, 1,829 patients with newly diagnosed colorectal cancer (colon cancer 833, rectal cancer 996), and 1,700 population-based controls. All enrolled patients were pathologically confirmed by Chonnam National University Hwasun Hospital between April 2004 and June 2008. Cases with secondary or recurrent tumors were excluded. The tumor stages were classified according to the TNM classification, including clinical or pathological TNM stages. Gastric cancer was classified by anatomical site as cardia (C16.0) or non-cardia (C16.1-16.8) and by histological types such as intestinal, diffuse, or mixed type.

The control group(n = 1,700) consisted of participants in the Thyroid Disease Prevalence Study [18], conducted from July 2004 to January 2006 in Yeonggwang and Muan Counties of Jeollanam-do Province and in Namwon City of Jeollabuk-do, Korea. At the time of their peripheral blood collections, all case and control subjects provided their informed consent to participate in this study. This study was approved by the Institutional Review Board of the Chonnam National University Hwasun Hospital in Hwasun, South Korea.

### Genotyping

Genomic DNA was extracted from peripheral blood using a QIAamp DNA Blood Mini Kit (Qiagen, Valencia, CA, USA), according to the manufacturer's protocol. Genotyping was performed by PCR-RFLP or real-time PCR. The genotyping protocol for PCR-RFLP was adapted from Frosst et al [19]. After HinfI (Takara, Tokyo,Japan) restriction enzyme digestion, samples were run on a 10% polyacrylamide gel (19:1) using Microtitre Array Diagonal Gel Electrophoresis (MADGE; MadgeBio, Grantham and Southampton, UK).

Genotyping by real-time PCR was performed by allelic discrimination, using dual-labeled probes containing locked nucleic acids (LNA), in a real-time polymerase chain reaction (PCR) assay. PCR primers and LNA probes were designed and synthesized by Intergrated DNA Technologies (IDT) (Coralville; City, IA, USA). PCR primers producing a 104-bp amplicon were as follows: forward primer, 5'-CTTTGAGGCTGACCTGAAGC-3' and reverse primer, 5'-TCACAAAGCGGAA GAA TGTG-3'. Dual-labeled LNA hybridization probes were 5'- FAM -ATG GcT ccc-BHQ1- 3' for the C allele and 5'- cy5-cgA CTc cCg C-BHQ2-3' for the T allele (LNA bases are denoted in upper case, single nucleotide polymorphisms are underlined). Real-time PCR was performed using a Rotor-Gene 3000 multiplex system (Corbett Research, Sydney, Australia) in a 10-μL reaction volume containing 200 nM PCR primer, 10-10 nM each probe, 0.5 U f-taq polymerase (Solgent, Daejeon, Korea), and 40 ng of genomic.

### Statistical analysis

The statistical significance of the differences between the patient and control groups was estimated by logistic regression analysis. Adjusted odds ratios (OR) were calculated with logistic regression model that controlled for sex and age and are given with 95% confidence intervals (CI). Subjects with the wild type genotypes (MTHFR 677 CC) were considered to be baseline risk. The expected frequency of control genotypes was checked by the Hardy-Weinberg equilibrium test. Interactions of genotype with smoking, alcohol consumption, and age were estimated using the logistic regression model, products of scores for smoking habit (0, never and 1, ever), drinking habit (0, non-drinker and 1, drinker), age (0, ≤ 65 years and 1, >65 years) and genotype (0, CC genotype for reference allele; 1, CT genotype and 2, TT genotype). Subgroup analysis was conducted on anatomical site, histological type, and TNM staging. The subjects for which there was missing data for smoking, drinking, anatomical site, histological type, and TNM staging were excluded in interaction and subgroup analysis related with these variables. All analyses were performed using the Statistical Package for the Social Sciences software version 17.0 (SPSS, Chicago, IL, USA).

## Results

The characteristics of the study population are presented in Table 1. The mean age of patients with gastric cancer and colorectal cancer was significantly higher compared with the control group. A statistically significant sex difference was also found between patients with gastric and colorectal cancer and healthy controls, and the control group had more female subjects. The proportion of smokers in gastric cancer cases was higher than that in the controls, but the proportion among colorectal cancer cases was lower than that in the controls. The proportion of drinkers in both cancer groups was lower than that in the controls.

**Table 1:** General characteristics of subjects

Characteristics	Controls	Gastric cancer	Colorectal cancer
No	1700	2213	1829
Age, mean,years	52.2 ± 14.3	60.2 ± 12.1*	61.9 ± 11.4**
≤ 65 years	1321(77.7)	1314(59.4)	985(53.9)
>65 years	379(22.3)	899(40.6)*	844(46.1)**
Sex			
Male	821(48.3)	1510(68.2)	1149(62.8)
Female	879(51.7)	703(31.8)*	680(37.2)**
Smoking habitat			
Never	1000(58.8)	1127(50.9)	1137(62.2)
Ever	655(38.5)	997(45.1)*	582(31.8)**
missing	45(2.6)	89(4.0)	110(6.0)
Drinking habitat			
Non-drinker	825(48.5)	1198(54.1)	1084(59.3)
Drinker	833(49.0)	921(41.6)*	623(34.1)**
Missing	42(2.5)	94(4.3)	122(6.7)
TNM Stage			
I		1138(51.4)	291(15.9)
II		305(13.8)	547(29.9)
III		290(13.1)	615(33.6)
IV		386(17.4)	230(12.6)
Unspecified stage		94(4.2)	147(8.0)
Tumor site			
Gastric cancer			
Cardiac		106(4.8)	
Non-cardiac		2093(94.6)	
Unspecified site		14(0.6)	
Colorectal cancer			
Colon			833(45.5)
rectum			996(54.5)
Histological type			
Intestinal		1286(58.1)	
Diffuse		561(25.4)	
Mixed		240(10.8)	
Unspecified type		126(5.7)	

The data is expressed as number, percent and mean ± standard deviation.

* Gastric cancer compared with control, *p *< 0.05;

**Colorectal cancer compared with control, *p *< 0.05.

Table 2 shows genotype distributions for MTHFR C677T and their adjusted odds ratios and 95% confidence intervals in gastric and colorectal cancer. The distribution of the MTHFR C677T gene polymorphisms in the controls was in Hardy-Weinberg equilibrium. The MTHFR C677T frequencies of CC, CT, and TT genotypes were 35.2%, 47.5%, and 17.3% among gastric cancer, 34%, 50.5%, and 15.5% in colorectal cancer, and 31.8%, 50.7%, and 17.5% in the controls, respectively. The frequencies of the C and T allele were 59.0% and 41.0% among gastric cancer, 59.2% and 40.8% in colorectal cancer, and 57.1% and 42.9% in the controls, respectively. Compared with the CC genotype, the TT genotype was significantly correlated with a reduced risk of colorectal cancer when adjustments were made for age and sex (age- and sex-adjusted OR = 0.792; 95% CI = 0.638-0.984, P = 0.035). Although our results for MTHFR 677TT and gastric cancer risk did not reach statistical significance, the observed trend (overall TT versus CC OR = 0.877; 95% CI = 0.719-1.070) indicates a somewhat protective effect. Whereas the 677CT genotype was significantly associated with a reduced risk of gastric cancer, the age- and sex-adjusted OR was 0.810 (95% CI = 0.696-0.942, P = 0.006).

**Table 2:** MTHFR C677T genotype distributions and adjusted odds ratio for gastric and colorectal cancer

				Gastric cancer		Colorectal cancer	
				
MTHFR C677T	Controls	Gastric cancer	Colorectal cancer	OR ^a ^(95% CI)	P value	OR ^a ^(95% CI)	P value
**CC**	540(31.8)	778(35.2)	622(34.0)	1		1	
**CT**	863(50.7)	1052(47.5)	923(50.5)	0.810(0.696-0.942)	0.006	0.923(0.787-1.082)	0.321
**TT**	297(17.5)	382(17.3)	284(15.5)	0.877(0.719-1.070)	0.195	0.792(0.638-0.984)	0.035
**C**	1943(57.1)	2608(59.0)	2167(59.2)	1		1	
**T**	1457(42.9)	1816(41.0)	1491(40.8)	0.915(0.832-1.008)	0.071	0.902(0.814-0.999)	0.047

OR ^a^, odds ratio adjusted for age and sex; CI, confidence interval;

Table 3 shows interaction between MTHFR C677T polymorphisms and smoking and drinking habit and age for gastric and colorectal cancer risk. When we used the MTHFR 677CC genotype as the reference, smoking habit, drinking habit, and age did not modify the association between the MTHFR C677T genotypes and the risk of gastric cancer or colorectal cancer. When results were stratified by anatomical site, histological type, and TNM staging, we observed no statistically significant differences in genotype distribution (Table 4).

**Table 3:** Interaction between MTHFR C677T polymorphisms and smoking and drinking habit and age for gastric and colorectal cancer risk.

	CT vs CC*	TT vs CC*	*p *for interaction^a^
		
	OR^a^(95%CI)	OR^a^(95%CI)	
**gastric cancer**			
Smoking habit			
Never	0.874(0.693-1.102	0.984(0.722-1.339)	
Ever	0.776(0.632-0.952)	0.825(0.630-1.079)	0.680
Drinking habit			
Non-drinker	0.801(0.651-0.986)	0.853(0.647-1.124)	
Drinker	0.827(0.656-1.042)	0.959(0.707-1.299)	0.847
Age			
≤ 65 years	0.852(0.711-1.020)	0.921(0.726-1.167)	
>65 years	0.700(0.529-0.925)	0.765(0.530-1.103)	0.581
**colorectal cancer**			
Smoking habit			
Never	0.993(0.762-1.293)	0.955(0.670-1.363)	
Ever	0.851(0.688-1.053)	0.666(0.499-0.888)	0.301
Drinking habit			
Non-drinker	0.854(0.689-1.059)	0.712(0.532-0.954)	
Drinker	0.950(0.735-1.229)	0.878(0.620-1.243)	0.635
Age			
≤ 65 years	0.966(0.819-1.211)	0.810(0.620-1.059)	
>65 years	0.789(0.597-1.043)	0.759(0.525-1.099)	0.361

OR^a ^,adjusted for age and sex; CC*, *CC *as reference group

Interaction^a ^was modeled as a product of smoking habit (0, never and 1, ever),

drinking habit (0,non-drinker and 1, drinker), age (0, ≤ 65 years and 1, >65 years) and genotype in score (0, *CC *genotype for reference allele; 1, *CT *genotype and 2, *TT *genotype)

**Table 4:** Subgroup analysis by TNM stage, tumor site and histological type for the MTHFR C677T polymorphisms.

	CT vs CC*	TT vs CC*
	
	OR^a ^(95%CI)	OR^a ^(95%CI)
**Gastric cancer**		
TNM stage		
I+II	0.810(0.685-0.958)	0.933(0.750-1.161)
III+IV	0.844(0.685-1.040)	0.792(0.597-1.050)
Tumor site		
Cardiac	0.906(0.569-1.442)	0.967(0.531-1.760)
Non-cardiac	0.811(0.696-0.944)	0.876(0.717-1.070)
Histological type		
Intestinal	0.815(0.680-0.978)	0.865(0.681-1.099)
Diffuse	0.832(0.672-1.031)	0.868(0.654-1.153)
Mixed	0.827(0.600-1.139)	1.111(0.748-1.652)
**Colorectal cancer**		
TNM stage		
I+II	0.947(0.775-1.158)	0.854(0.652-1.119)
III+IV	0.823(0.679-0.997)	0.667(0.510-0.871)
Tumor site		
Colon	0.942(0.744-1.146)	0.727(0.553-0.952)
Rectum	0.888(0.636-1.073)	0.855(0.666-1.099)

OR^a^, adjusted for age and sex; CC*, CC as reference group

## Discussion

The current study represents the largest sample (2,213 gastric cancer, 1,829 colorectal cancer, and 1,700 controls) of the Korean population ever used to evaluate the possible association between the MTHFR C677T gene polymorphisms and susceptibility to gastric and colorectal cancer. The T allele was found to provide a weak protective association with gastric and colorectal cancer.

Previous reports on the MTHFR polymorphism and their associations with stomach cancer have been quite inconsistent. Of the published studies, some studies especially in China [20-22], Italy[9], and Mexico[10], found that the MTHFR 677TT genotype was a strong risk factor for gastric cancer, others no association[15-17], and only one suggested a decreased risk[23]. Although our results for MTHFR 677TT and gastric cancer risk did not reach statistical significance, the observed trend (overall TT versus CC OR = 0.877; 95% CI = 0.719-1.070) indicates a somewhat protective effect, whereas the combination of MTHFR 677CT revealed a significant protective association with gastric cancer, the OR of overall CT versus *CC *was 0.810 (95% CI, 0.696-0.942). In a Mexican population, Galvan-Portillo et al. [23] reported a significant reduction in diffuse gastric cancer risk for the MTHFR 677 TT genotype among individuals with high consumption of folate (OR = 0.23; 95% CI 0.06-0.84) compared to wild-type homozygous and heterozygous genotypes combined. In fact, in other malignancies such as acute lymphocytic leukemia[24,25], breast cancer[26], and colorectal cancer[27-29], studies have also reported a protective association between the MTHFR 677TT genotype and the risk of some cancer. In addition, Jiang et al. [30] also suggested that individuals with adequate folate status who are homozygous for the MTHFR 677TT polymorphism have reduced the risk of colorectal cancer. Furthermore, Chen, et al. [31] observed that the TT genotype was protective in folate-replete subjects, whereas the combination of TT and low folate status conferred no protection, or even showed an increased risk. These results suggest that the cancer risk associated with MTHFR polymorphisms may exhibit a gene-nutrient interaction that depends on the level of folate intake or plasma folate levels. However, we could not evaluate the gene-nutrient interaction in our study due to a lack of data regarding the plasma folate levels of case group. Although we had information on the plasma folate levels of 1,700 healthy individuals in the control group, which was based on the general population. The median level of plasma folate was 22.7 nmol/L in our controls. Hao et al. [32] reported that the median values of plasma folate were 16.7 nmol/L in South China and 8.4 nmol/L in North China. This implies that Korean populations might have a relatively higher plasma folate level than do Chinese people. It may be partially explained by the dietary habits of Koreans. Rapid economic growth has led to changes in Korean food consumption patterns: consumption of the staple foods such as rice, barley and potatoes has declined, whereas consumption of meat, fruit, vegetables and dairy products has increased. According to Korean National Health and Nutrition Examination Survey report in 1998, 2001 and 2005, increasing trends in daily vegetable consumption were shown in Korea[33]. It has been known that vegetables and fruits are major source of folate. In addition, the frequency of MTHFR *TT *homozygotes was 17.5% in our 1,700 healthy controls, which was consistent with findings for the control subjects in Japan (17.2%)[34], but was lower than that reported for control subjects in China (31.1-41%)[22,35]. Compared to Chinese people, the Korean population might have a relatively low frequency of the MTHFR 677TT genotype and a relatively high plasma folate level. This might provide a partial explanation why the MTHFR 677 mutations were found to be protective for gastric and colorectal cancer in our study.

With regard to the MTHFR C677T genotype and the risk of colorectal cancer, although it has been proposed that the MTHFR polymorphism might be involved in the etiology of cancer through regulation of DNA synthesis and repair, some subsequent studies have not provided evidence for their association with colorectal cancer. In a Japanese case-control study of colorectal cancer[36,37], results showed that the MTHFR C677T polymorphism did not have a role in the development of colorectal cancer. In addition, Zeybek et al. [17] in Turkey, Plaschke et al. [38] in Germany, and Derwinger et al. [14] in Sweden also reported no association between the risk of colorectal cancer and the MTHFR 677TT genotype. However, several studies observed positive associations between the MTHFR 677TT genotypes and increased risk for colorectal cancer. Miao et al. [39] in China and Guerreiro et al[40]in Portugal demonstrated that the MTHFR 677TT presented a increased risk of colorectal cancer. Our study is consistent with a recent meta-analysis that concluded a small but significant protective effect of MTHFR C677T exists against colorectal cancer risk (overall TT versus CC OR = 0.93; 95% CI, 0.89-0.98) for a worldwide population [28].

Considering that heavy drinking and smoking are recognized as risk factors for gastric and colorectal cancer, we included these factors in our study, our findings showed no interactions between the MTHFR C677T polymorphisms and drinking and smoking. With respect to alcohol consumption and MTHFR genotypes, the results were inconsistent. For colorectal risk, earlier studies reported that a protective effect of the MTHFR TT genotype disappeared in those with a high alcohol intake [27,29]. A recent Chinese study showed a 5-fold increased gastric cancer risk in drinkers with the MTHFR677T/T genotype[41], whereas a Japanese study showed that the MTHFR 677TT genotype reduced esophageal cancer risk among heavy-drinkers[42]. In addition, no interactions were found between the MTHFR C677T polymorphism and alcohol consumption in the risk of gastric cancer [10,43]. Cigarette smoking may decrease folate in plasma and produce a localized deficiency of folic acid. Boccia et al. [44] observed that ever smokers carrying the MTHFR 677 T allele showed a significant increased risk of gastric cancer. Our results showed no interaction between MTHFR C677T and smoking in the risk of gastric and colorectal cancer. Yang et al. [42] also did not observe any interaction between the effect of the MTHFR C677T polymorphisms on esophageal cancer risk and smoking.

To the best of our knowledge, only two studies have examined the MTHFR polymorphisms and the risk of gastric cancer and colorectal cancer in a Korean population. However, those studies were based on limited sample sizes. One study by Kim et al. [15] involving 133 gastric cancer and 445 controls showed no relationship between the MTHFR genotype and gastric cancer. Another study included 243 colorectal cancer and 225 controls, results from this study showed no relationship between the MTHFR C677T genotype and the overall risk of colorectal cancer, but the T allele was found to be associated with an increased risk of colon cancer and with a somewhat decreased risk of rectal cancer[45]. Our result showed a tendency for lowered colorectal cancer risk in individuals with the MTHFR 677TT genotype and the same tendency was also found in colon and rectal cancer.

The conflicting results regarding the associations between MTHFR C677T polymorphisms and risks for gastric cancer and colorectal cancer may be due to different ethnicities, different subtypes, and differences in regional dietary and local carcinogen exposures. In addition, many previous studies have considered relatively small populations, leading to difficulties in assessing the true statistical significance of the data.

The limitations of our study are that we did not determine serum folate levels or dietary folate intake in the case groups, and we also did not collect the detailed data on the risk factors of gastric cancer and colorectal cancer. Therefore, we cannot add to the debate on the relationship between gene-environment interactions.

## Conclusions

The present case-control study in Korea found a protective effect of the MTHFR C677T variant genotype for gastric and colorectal cancer and suggested that the effects of the MTHFR C677T genotype may differ in populations with different levels of folate intake.

## Competing interests

The authors declare that they have no competing interests.

## Authors' contributions

MHS planned the analysis. CLH performed the majority of experiments, participated in the study design and drafted the manuscript. HNK and HRS participated in the experiments. JMP performed data analysis. HJS, JEH and SHK provided clinical material. SSK, JSC, HRK and YKP participated in its design and coordination. All authors read and approved the final manuscript.

## Pre-publication history

The pre-publication history for this paper can be accessed here:

http://www.biomedcentral.com/1471-2407/10/236/prepub
